# Why Not Pedal for the Planet? The Role of Perceived Norms for Driver Aggression as a Deterrent to Cycling

**DOI:** 10.3390/ijerph20065163

**Published:** 2023-03-15

**Authors:** Laura S. Fruhen, Patrick Benetti, Lisette Kanse, Isabel Rossen

**Affiliations:** 1 School of Psychological Science, The University of Western Australia, Crawley, WA 6009, Australia; 2 StudySmarter, The University of Western Australia, Crawley, WA 6009, Australia

**Keywords:** spillover, green psychological workplace climate, norms, workplace, road safety, sustainability

## Abstract

Cycling has many benefits for humans and the planet. This research investigates perceived norms and driver behavior toward cyclists as issues that may be useful for addressing reluctance to cycle. It connects perceived norms observed in the road context regarding aggressive driver behavior towards cyclists, and norms observed in workplaces regarding sustainability (perceived green psychological workplace climate) with driver aggressive behavior toward cyclists. Self-reported online survey responses from *N* = 426 Australian drivers were collected. Perceived norms regarding aggressive driver behavior toward cyclists were linked to drivers engaging more frequently in such behavior, but no such link was found for perceived green psychological workplace climate. However, perceived green psychological workplace climate moderated the link between perceived norms regarding aggressive driver behavior toward cyclists and drivers engaging in such behavior. When drivers perceived aggression toward cyclists to be common on the road, perceived green psychological workplace climate weakened the link between perceived norms regarding aggressive driver behavior towards cyclists and drivers engaging in such behavior. Findings reinforce the role of perceived road context norms regarding aggressive driver behavior toward cyclists for drivers engaging in such behavior. They illustrate that, while not directly linked, sustainability norms perceived in other contexts have a role in shaping car driver behavior towards cyclists. The study’s findings suggest that interventions targeted at aggressive behavior toward cyclists in road contexts can focus on driver behavior norms and can be complemented by normative interventions in other settings to shape a key deterrent to cycling.

## 1. Introduction

Cycling as an active mode of transport is a key option to address many environmental and public health issues that arise from increased pressures on our planet. It has been shown to have health benefits for cyclists themselves [1,2,3], to reduce traffic congestion and pollution [4,5,6,7,8], and to have monetary benefits for cyclists and the wider economy [9]. As such, cycling can address the two key pillars that characterize planetary health: the health of human civilization and that of the natural environment [10,11]. Despite these benefits, research has shown that cycling participation rates continue to remain low in many countries [12]. For instance, the European Commission [13] reported that 50% of citizens indicated that they never use a bicycle for transportation. Further, of those who did cycle, only 8% stated that it was their most common mode of transport. In Australia, where the current study is based, the percentage of the population reported to cycle regularly each week is at comparatively low levels [14] and has decreased from 15.5% in 2017 to 13.8% in 2019 [15]. 

One key deterrent to cycling is its perceived [16] and actual risks. Indeed, Australian and international accident statistics show increasing cyclist deaths (~4% of road-user fatalities) and injuries (~15% of road-user hospitalizations) over the last decade [17,18,19,20]. To make cycling more accessible, reducing both real and perceived risk is a key strategy. Accident statistics show that most cyclist casualty crashes involve motor vehicles [19]. Cyclists also report that most of the aggressive behavior that they experience on roads comes from motorists [21] and this type of behavior from motorists is a key deterrent from cycling [16].

These statistics suggest that addressing motorists’ aggressive behavior towards cyclists on the roads is central to making cycling safer and more popular. Aggressive behavior towards cyclists considered in the literature (i.e., hand gestures and verbal abuse [22]) represent aggressive warnings [23]. Such behaviors are a likely aspect of car drivers’ and cyclists’ interactions that may contribute to cycling deterrence. Aggressive warnings, while not severe per se, are more common than more severe aggressive behaviors and can contribute to an overall environment that is perceived as hostile towards cyclists (as has been identified to be the experience of cyclists in Australia [24]). These behaviors may also be leading indicators of more severe offenses toward cyclists. Perceived social norms (i.e., unwritten, or unspoken rules that guide behavior [25]) are particularly critical for aggressive warnings as laws cannot effectively regulate them [26], making reliance on unspoken rules (i.e., norms) important. Rooted in the Theory of Planned Behavior [27] researchers have taken steps toward understanding the role of perceived social norms in shaping car driver behavior on the roads. Parker et al. [28] identified that social norms influence whether drivers are likely to cut across traffic to leave a motorway or overtake other vehicles on the inside. Björklund and Åberg [29] reported that social norms (other drivers’ behavior and the width of the road) influenced the behavior of drivers at traffic intersections. Bingham et al. [30] reported that risk-related norms conveyed by a more or less risk-averse passenger were linked to male teen drivers intentionally engaging in risky driving behaviors (e.g., traffic violations). Of specific relevance to our research, one study [22] showed drivers’ perceived social norms regarding how often other drivers show aggressive behavior toward cyclists to be linked with their engagement in such aggressive behavior toward cyclists.

To extend research on the role of norm perceptions for drivers’ aggressive behavior toward cyclists, this paper considers norm perceptions that occur in two different contexts: the road context, and an adjacent but separate context, namely the workplace. The perceived norm specific to the road context is directly concerned with aggressive behavior towards cyclists; and the workplace-related perceived norm concerns a related issue, sustainability, which is aligned with cycling’s environmental benefits. We consider these two perceived norms to identify possible spillover effects in the ways in which they operate together. In other words, we argue that road behavior does not occur in a vacuum and what happens in other contexts may be relevant to explaining behavior on the roads. We focus on work-related norms because most driving occurs during commutes in Australia where this study is set [31] and experiences from work can spill over into other domains [32]. The focus on sustainability in the norm perception outside of the road context was chosen because cycling is seen as a sustainable practice [3]. This study tests the direct links of these two perceived norms with driver behavior, as well as the extent to which they interact with one another in connection to driver behavior.

### Norm Content and Context

To extend what is already known about social norms and drivers’ aggressive behaviors toward cyclists [22], the current study brings together perceived norms that differ in content and the context in which they occur. First, we consider drivers’ norm perceptions that are specific to behavior towards cyclists and occur in road settings. Second, we consider norms that target sustainable behaviors (i.e., green psychological workplace climate [33]) as related norm content and that occur outside of the road context, namely at work. Perceived social norms, while locally rooted, may not be limited in their influence on one specific context. Exploring the effects of these two norms can unlock further understanding of the ways in which different types of norms, from separate but related contexts, operate together in shaping behavior. Importantly, doing so can provide guidance for behavior change strategies focused on changing norm perceptions. When it comes to undesirable behavior, norm interventions directly targeting a behavior can backfire and lead to an increase in such behavior. For example, if it is communicated that aggression toward cyclists is a common behavior that warrants attention, with the intention of alerting people to the issue, this information may unintentionally lead people to perceive the behavior as common, and therefore normal and justified. This unintended effect has been labeled a boomerang effect [3,34]. Priming people toward a certain issue can further affect how norms shape behavior [35]. Targeting norms (i.e., regarding sustainability rather than cyclists directly) that are adjacent (i.e., located in the workplace) yet related to driver behavior toward cyclists (seeing that cycling is considered a sustainable behavior [3]), has the potential to overcome these pitfalls.

The first norm considered in this study is specific to cyclists’ and car drivers’ interactions on the roads. Previous research has shown that perceived norms regarding the frequency of aggressive driving behavior toward cyclists (i.e., based on perceived aggressive behavior of other drivers toward cyclists) relate to car drivers reporting aggressive behavior toward cyclists in the United Kingdom [22]. The United Kingdom and Australia share many similarities culturally [36]. However, they differ in their levels of car ownership, average speed limits in residential and heavy traffic areas, and government support for cycling, all of which have been shown to impact behaviors on the road (e.g., cycling participation [14]). Replicating the link between perceived norms regarding aggressive driver behavior toward cyclists with aggressive driver behavior toward cyclists in the Australian context can illustrate the robustness of the effect of these norms on driver aggressive behavior toward cyclists. We hypothesize:

**Hypothesis** **1.**  *Perceived norms regarding aggressive driver behavior toward cyclists are positively linked with self-reported aggressive driver behavior toward cyclists.*

The second norm considered in this study is set in the workplace and captures perceptions of norms related to sustainability at work via a concept labeled green psychological workplace climate [33,37]. Green psychological workplace climate has been defined as employees’ perceptions and interpretations of their organization’s environmental sustainability policies, procedures, and practices [33,37]. It is positively connected to pro-environmental behaviors at work (e.g., saving water, conserving energy, recycling, and avoiding waste [38,39]) making it relevant to cycling, which is often seen as a sustainable behavior [3].

Some attributes of workplace norms and the context in which they are encountered make this proposed spillover of their influence into traffic settings more likely. Colleagues are key social influences [40] and work is a core aspect of people’s lives, with many spending a third of each weekday at work, meaning exposure to workplace norms is considerable. Workplace norms are a core aspect of workplaces and represent “shared perceptions of organizational policies, procedures and practices both formal and informal” (i.e., represented in the concept of workplace climate [41] (p. 22)). These shared perceptions provide a frame of reference for appropriate behavior and have been identified as influencing behavior [41]. The effect of these norms may expand beyond the workplace itself, as workplaces have been shown to influence behavior in other domains (i.e., the home [32]). The perceived norms’ effect is likely to spill over to the driving context given that a substantial amount of driving occurs adjacent to work during commute time (the average weekly commute time in Australia was 4.5 h in 2017 [31]). When commuting, workplace norms may impact behavior because exposure has been recent and the two contexts are often seen as interconnected as is evident in the language that we use to describe the activity (i.e., commuting to/from work). Working in an environment that values sustainability may make cycling as a sustainable practice more salient and mark it out as aligned with the norms perceived at work. Consequently, those working in such environments may value cyclists more and show less aggressive behavior toward them. We hypothesize:

**Hypothesis** **2.**  *Perceived green psychological workplace climate is negatively linked with self-reported aggressive driver behavior toward cyclists.*

When perceived norms operate together, behavior is most likely to be impacted when they are aligned [35,42,43,44]. When inconsistencies between perceived norms occur (i.e., one is supportive of a behavior and one is not) the effect of the supportive normative message is diminished, resulting in reduced occurrences of the targeted behavior [44,45]. Similarly, a supportive norm can counteract the effect of a destructive one [46].

Regarding the two perceived norms considered in this study, we propose that they operate in the same way. A more positively perceived green psychological workplace climate may counteract the impact of perceived norms regarding drivers’ aggressive behavior toward cyclists on the behavior itself. Specifically, a higher perceived green psychological workplace climate may activate a frame of reference toward cycling that recognizes it more readily as a pro-environmental behavior rather than primarily activating stigma associated with cycling [47], resulting in a less amplified link between the perceived descriptive norm of driver aggressive behavior toward cyclists and aggressive driving behavior toward cyclists. When drivers are exposed to lower levels of green psychological workplace climate, and at the same time perceive descriptive norms regarding driver aggressive behavior toward cyclists to be high, they show more aggressive behavior toward cyclists. This effect is likely to occur given that drivers perceiving the norms in this way would not recognize the pro-environmental aspects of cycling as being valued by their colleagues and perceive aggression toward cyclists to be legitimized on the roads by their perception that this behavior is common. It is further predicted that this moderating effect is less pronounced when norms regarding driver aggressive behavior toward cyclists are perceived to be low. Accordingly, we hypothesize:

**Hypothesis** **3.**  *Perceived green psychological workplace climate moderates the link between perceived norms regarding aggressive driver behavior toward cyclists and self-reported aggressive driver behavior toward cyclists, so that as perceived green psychological workplace climate increases, this link weakens.*

**Figure 1:**
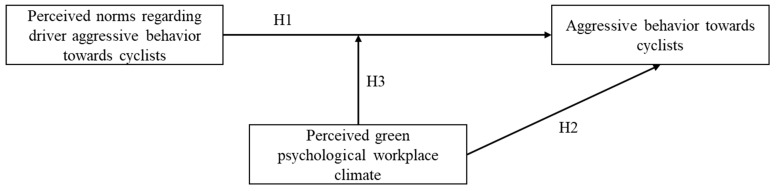
Norms and aggressive driving behavior toward cyclists.

Our hypotheses are summarized in the research model represented in Figure 1 below.

## 2. Materials and Methods

### 2.1. Participants

The sample (it should be noted that the dataset reported here is a subset of a dataset previously reported on by Fruhen et al. (2021) on a separate research question) consists of (*N* = 426) participants who completed an online survey (for a detailed breakdown of the sample characteristics please refer to Table S1 in the Supplementary Materials). All participants were screened so that the sample only included those whose survey responses were complete, who had indicated that they currently work, held a driver’s license, and live in Australia. Men (*N* = 206) and women (*N* = 212) were represented almost equally in the sample (8 participants selected not to indicate their gender). Participants’ age ranged from 18 years to 82 years, with a mean age of 43.59 years (*SD* = 13.59). On average, participants had worked for their current organization for 9.99 years (*SD* = 9.06), with the majority reporting they worked full-time (66.9%, *N* = 285). Participants reported working in a range of industries (classified using the Australian Bureau of Statistics, 2006), with education and training (18.1%, *N* = 77), professional, scientific, and technical services (13.1%, *N* = 56) and others (12.2%, *N* = 52) being among the most common. The composition of our sample (as seen above) regarding industry was similar to that reported by the ABS (2019) industry statistics so it is reasonably representative of the Australian working population. The time participants had held their driver’s license ranged from 1 year to 65 years (*M* = 25.590, *SD* = 13.594). Most participants indicated that they had regularly ridden a bicycle on the road since the age of 12 (66.0%, *N* = 281); however, only 34.5% of participants reported regularly (at least once a week) riding a bike in the last 12 months.

Participant inclusion was based on convenience sampling. Drawing on the Australian Government’s Bureau of Infrastructure, Transport and Regional Economics estimate of the number of license holders in Western Australia for the year in which the data were collected (2020; the estimated number of license holders = 2,027,379; https://www.bitre.gov.au/sites/default/files/is_084.pdf, accessed on 28 February 2023), the sample included in this study is representative of the population of Western Australian license holders (minimum representative sample is n = 385; CI 95% and margin of error 5%).

### 2.2. Measures

Perceived norms regarding aggressive driver behavior toward cyclists were measured with an adapted version of the aggressive warning subscale of the Driver Anger Indicator Scale (DAIS [22,23]). The scale captures perceived norms regarding aggressive warnings (e.g., rude hand gestures or honking the horn [22]). It includes six items that ask participants to indicate on a scale of 1 (never) to 5 (very often) how frequently they perceive other drivers to engage in such behaviors toward cyclists. An example item is: indicate how often other drivers would engage in the following behavior toward a cyclist: …swear at them. Cronbach’s alpha for the scale was α = 0.92 and a mean score across all items was computed.

Perceived green psychological workplace climate was assessed with five items by Norton et al. [39]. These items capture perceptions of the priority that a workplace provides to sustainability policies, procedures, and practices. An example item is: my company is worried about its environmental impact. Participants rated each item using a 5-point scale, ranging from 1 = strongly disagree to 5 = strongly agree. Cronbach’s alpha for the scale was α = 0.92 and a mean score across all items was computed.

Self-reported aggressive driving behaviors toward cyclists were measured with the Fruhen and Flin [22] adaption of the aggressive warning subscale of the DAIS. The measure included the same six items as the perceived driver aggression norm scale to assess driver behavior. However, in this case, the scale asked how often the participants themselves engaged in each of the driver behaviors toward cyclists. Responses were recorded using a response scale ranging from 1 = never to 5 = very often. An example item is: indicate how often you would engage in the following behavior toward a cyclist:… cut off. Cronbach’s alpha for the scale was α = 0.81 and a mean score across all items was computed.

Control variables included demographic information related to participants’ gender, own cycling frequency in the past 12 months (ranging from 1 = every day to 6 = never, so that a higher number indicates a lower frequency [48]), how long they had held their driver’s license, how long they had been in their job, and whether they were employed full-time or part-time. Gender and own cycling frequency represent possible confounding variables related to driver aggressive behavior, and as such, were controlled for [49,50]. How long participants had held their driver’s license, their job tenure, and whether they were in full- vs. part-time employment, were controlled for, as these represent proxies of exposure to the contexts to which the norms being investigated relate. Such exposure has been shown to influence perceptions of norms [51].

Further, negative attitudes toward cyclists were also included as a control variable to capture the extent to which the effects of norm perceptions go above and beyond such attitudes, given that they have been shown to be linked with aggressive behavior toward cyclists [22,50,52]. Ten items by Rissel et al. [48] were used to measure this concept. An example item is: it is very frustrating sharing the road with cyclists, the response scale ranged from 1 = strongly disagree to 5 = strongly agree. Cronbach’s alpha for the scale was α = 0.89 and a sum score across all items was computed.

### 2.3. Additional Details Included in the Survey

To account for possible differences in driver responses to different types of cyclists, participants were shown a picture of different cyclist types and responded to the survey questions regarding aggressive driving behavior toward cyclists and norms regarding such behavior in reference to the cyclist shown: a male in lycra, a male in casual attire, a female in lycra, or a female in casual attire. A between-subjects design was used whereby a participant was shown a cyclist picture randomly drawn from these four types. Comparisons via the Mann–Whitney-U test were carried out to identify the extent to which participant responses varied depending on the shown cyclists’ gender and attire (note that Kolmogorov–Smirnov tests indicated responses for aggressive driving behavior toward cyclists and norms regarding such behavior were non-normally distributed, so that non-parametric tests were used for this analysis) (*D*_aggressive driving behavior toward cyclists_ (425) = 0.35, *p* < 0.001; *D*_perceived norms regarding aggressive driving behavior toward cyclists_ (425) = 0.11, *p* < 0.001). Comparison of the median reported values for aggressive driving behaviors toward cyclists between participants who were shown cyclists in lycra attire and those who were shown cyclists in casual attire showed that there was no variation in responses (*Md*_lycra attire_ = 1.00; *Md*_casual attire_ = 1.00; *U* (*N*_casual attire_ = 208, *N*_lycra attire_ = 217) = 24,444.50, *z* = 1.79, *p* = 0.07). Comparison of the median reported values for perceived norms regarding aggressive driving toward cyclists between participants who were shown cyclists in lycra attire and those who were shown cyclists in casual attire showed there was no variation in responses (*Md*_lycra attire_ = 2.67; *Md*_casual attire_ = 2.67; *U* (*N*_casual attire_ = 207, *N*_lycra attire_ = 218) = 23,013.00, *z* = 0.36, *p* = 0.72). Comparisons based on cyclists’ gender also showed no differences in aggressive driving toward cyclists (*Md*_female cyclist_ = 1.00; *Md*_male cyclist_ = 1.00; *U* (*N*_male cyclist_ = 214, *N*_female cyclist_ = 211) = 22,104.50, *z* = −0.45, *p* = 0.65) nor perceived norms regarding aggressive behavior toward cyclists (*Md*_female cyclist_ = 2.58; *Md*_male cyclist_ = 2.67; *U* (*N*_male cyclist_ = 213, *N*_female cyclist_ = 212) = 20,570.50, *z* = −1.60, *p* = 0.11). Accordingly, we analyzed responses regarding aggressive driving behavior toward cyclists and norms regarding such behavior across the different types of cyclists shown to the participants, irrespective of the gender or attire.

### 2.4. Procedure

The survey was hosted online on the university’s website. All responses were collected anonymously, and participation was voluntary. Information about the study and a link to the online survey were posted on social media platforms. Additionally, the study was promoted in an article published in a local newspaper, and in two local radio interviews.

### 2.5. Analysis

The data were analyzed using SPSS-27. The residuals of the outcome measure, aggressive behavior toward cyclists, were shown in P-P plots to be normally distributed. Accordingly, the main analysis used parametric statistics, namely Pearson correlation and regression analysis. The interaction effect was tested using model 1 of Hayes’ PROCESS Macro in SPSS [53]. The interaction was tested using unstandardized coefficients via bootstrapping procedures with 5000 resamples.

## 3. Results

Pearson correlations shown in Table 1 indicate a significant positive link between perceived norms of driver aggression toward cyclists and driver self-reported aggression toward cyclists (*r* = 0.38; *p* < 0.01). The correlation between perceived green psychological workplace climate and driver self-reported aggression toward cyclists was not significant (*r* = −0.07; *p* > 0.05).

Regression analysis (see Table 2) indicated that, together, the control variables explained 16.5% of the variance in aggressive driver behavior toward cyclists. Of note, attitudes toward cyclists’ link with aggressive behavior was significant (*β* = 0.38; *p* < 0.001). The addition of perceived norms of aggressive driver behavior toward cyclists and perceived green psychological workplace climate explained a further 13.9% of the variance. In support of Hypothesis 1, perceived norms of aggressive driver behavior toward cyclists were positively linked with self-reported aggressive driver behavior toward cyclists (*β* = 0.37; *p* = 0.000).

Perceived green psychological workplace climate was not linked with self-reported aggressive driver behavior toward cyclists (*β* = −0.03; *p* = 0.45). Accordingly, Hypothesis 2 was not supported.

Finally, Table 3 reports the results related to the interaction effect proposed in Hypothesis 3. The overall model explained 33% of the variance in aggressive driver behavior toward cyclists. The interaction term was significantly linked with aggressive driver behavior toward cyclists (effect = −0.07, CI 99% −0.12/−0.03).

**Figure 2:**
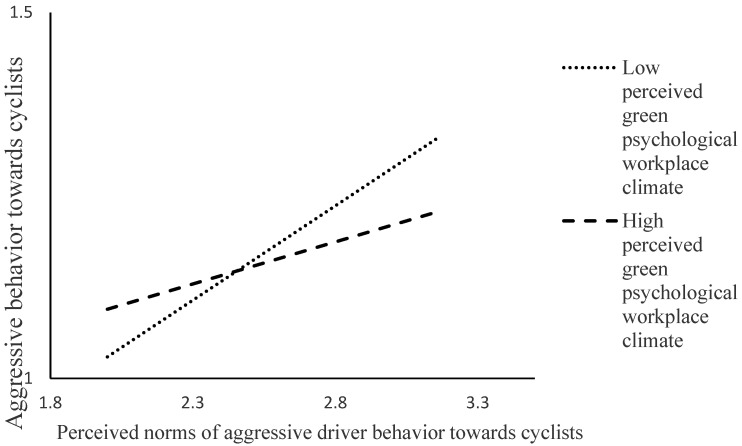
Interaction between perceived norms regarding driver aggression toward cyclists and perceived green psychological workplace climate.

The interaction effect is shown in Figure 2. The figure illustrates that the link of perceived norms regarding aggressive driving toward cyclists with more aggressive driving toward cyclists was stronger when perceived green psychological workplace climate was low rather than high. Drivers who reported low levels of perceived green psychological workplace climate and high levels of perceived norms of aggressive driver behavior toward cyclists reported the highest level of aggression toward cyclists. In contrast, drivers who reported high levels of perceived green psychological workplace climate and high levels of perceived norms of aggressive driver behavior toward cyclists on aggressive driver behavior toward cyclists showed a comparatively lower level of engagement in aggressive behavior toward cyclists. The relationship between perceived norms regarding aggressive behavior toward cyclists and aggressive behavior toward cyclists was significant for both high (effect = 0.12, CI 99% 0.05/0.18) and low perceived green psychological workplace climate (effect = 0.26, CI 99% 0.20/0.31).

Overall, these findings support the proposed weakening of the link between perceived norms regarding aggressive driver behavior toward cyclists and self-reported aggressive driver behavior toward cyclists as the value of green psychological workplace climate increases. Accordingly, Hypothesis 3 was supported.

## 4. Discussion

This study investigated the role of perceived social norms in shaping car drivers’ aggressive behavior toward cyclists, which is a key deterrent for cycling uptake. It considered two perceived norms that differ in content and context. The results indicate that both perceived norms had different and unique associations with aggressive driver behavior toward cyclists. First, in support of Hypothesis 1, perceived norms regarding aggressive behavior toward cyclists was positively related to driver self-reported aggressive behavior toward cyclists. Second, in contrast to our Hypothesis 2, perceived green psychological workplace climate was not found to have a direct link with driver self-reported aggressive behavior toward cyclists. Third, these workplace-related sustainability norms were found to affect drivers’ self-reported aggressive behavior toward cyclists more indirectly. Specifically, in support of Hypothesis 3, we found perceived green psychological workplace climate to moderate the link between perceived norms regarding aggressive driver behavior toward cyclists and self-reported aggressive behavior. The moderation was such that the link between perceived norms regarding aggressive driving toward cyclists with aggressive driving toward cyclists was stronger when perceived green psychological workplace climate was low, compared with when it was high.

Based on these findings, we identify two key contributions that this research makes to the knowledge of the ways in which perceived norms affect driver behavior toward cyclists. Such knowledge can be used to inform ways to target the prevalence of aggressive driver behavior toward cyclists, which can be a deterrent to the uptake of cycling as a healthy, sustainable practice. Overall, our findings indicate that it can be worthwhile to consider perceived social norms as one strategy that can complement other approaches to making roads more cycle friendly (e.g., cycle lanes, removing parking, adding trees [54]; and cycle route planners [55]). First, the link between perceived norms regarding driver aggression toward cyclists and driver aggressive behavior toward cyclists replicates previous findings of this link reported in the UK [22]. As such, this replication speaks to the consistency of this effect in the Australian context and suggests that across these two countries, it is robust. Accordingly, this study’s findings reinforce the important role that perceived driver norms regarding aggressive behavior toward cyclists play in explaining driver aggression toward cyclists and the importance of considering them when wanting to increase bike riding frequencies. Second, the study provides novel and refined insights into the role of norms outside of the road context in relation to driver behavior toward cyclists. Our results show that the spillover of these norms from work to the driving context that we had proposed does not occur directly. Of note, perceived green psychological workplace climate was indicated to have a subtle role in shaping car driver behavior toward cyclists on the roads. Specifically, the link between perceived norms regarding aggressive driving toward cyclists with aggressive driving toward cyclists was weakened when perceived green psychological workplace climate was high, compared with when it was low. Thus, in contexts in which perceived norms suggest to drivers that aggressive behavior toward cyclists is quite common, a strong perceived green psychological workplace climate can make drivers less likely to follow their norm perceptions regarding aggressive behavior toward cyclists. Australian roads are a context in which aggression and other negative behaviors toward cyclists are seen as not only common but also justified [56]. This finding speaks to the utility of norms in adjacent contexts to the roads in shaping driver aggression toward cyclists in Australia and other countries with a similar norm context.

The results align with research suggesting that a supportive norm can counteract a destructive one [46] and in this case extends this knowledge, as our results show this effect occurred across contexts and with a norm that is not explicitly concerned with the targeted behavior. When it comes to undesirable behavior that is somewhat common (as is the case for aggressive driver behavior toward cyclists in Australia [50]), descriptive norm interventions targeted at that specific behavior can backfire and lead to an increase in such behavior. As highlighted in the introduction communicating that aggression toward cyclists is a common issue may have an unintended boomerang effect [33,34,35], so that such behavior occurs more often following such communication. Targeting norms whose content is adjacent (i.e., located in the workplace and focused on sustainability rather than cyclists directly) yet related to driver behavior toward cyclists has the potential to overcome such issues and our results support this suggestion.

Of note, our findings also suggest that in contexts where perceived norms of aggression toward cyclists are low, high perceived sustainability norms in the workplace can contribute to drivers being more frequently aggressive toward cyclists compared with when perceived sustainability norms in the workplace are low. It is possible that in contexts where aggression toward cyclists is seen as uncommon, a workplace that prioritizes sustainability may contribute to a “too much of a good thing phenomenon”. In effect, it is possible that drivers in contexts where aggression toward cyclists is not seen as common and whose workplaces prioritize sustainability are over-sensitized toward sustainability-related issues, including cycling. Consequently, they likely experience the combination of low perceived norms regarding aggressive driver behavior toward cyclists and strong sustainability norms at work as “overdoing it” and when seeing a cyclist in traffic, act more negatively toward them.

### Limitations and Future Research

The study’s results need to be considered within the study’s limitations. First, we rooted our predictions of the links between norms and behavior in the theory of planned behavior [27]. It needs to be considered that despite this theoretical grounding, a definitive claim that norms “cause” behavior cannot be made. It is just as plausible that the perceived prevalence of driver aggression by others serves as a form of justification or validation of the participants’ own aggressive behavior.

Second, because both content (i.e., sustainability) and context (i.e., workplace) of the perceived norm studied outside of the road context were different from the targeted behavior (i.e., aggression toward cyclists on the roads), it is not clear to what extent either content or context may explain the effects of perceived green psychological workplace climate on aggressive driver behavior toward cyclists. Future research can test (workplace) norms, which target other types of content related to cycling, for example, physical activity [14]. Other ways in which workplace-focused norms relate to cycling may be conveyed can also be considered, such as the perceived number of cyclists in a company, or the availability of end of trip facilities. Further, future investigations into workplace norms can account for variation in their influence based on workers’ level of identification with their colleagues and their company. Research shows that social identification moderates the link of norms with behavior in other contexts [57] and may further amplify the role of workplace norms in relation to driver behavior toward cyclists.

Third, our study did not include any measure of reluctance to take up cycling or use a bicycle as a healthy sustainable mode of transport as an outcome variable and relies on findings from other studies (e.g., [16]) to make this link. Future studies into the role of road behavior and related norms should consider designs that include capturing bicycle use/uptake reluctance.

Furthermore, our study considered social norms as focal variables and controlled for attitudes, both of which are key antecedents of behavior stipulated in the theory of planned behavior [27]. However, our study did not consider intention, which has been identified as relevant to cycling mode choices [58]. Similarly, recent research shows social influence via social interactions to operate alongside social norms in informing travel mode choices [59]. Future research will benefit from expanding the here tested model and include these additional variables.

Finally, our study used self-reports of aggressive behavior toward cyclists, so that the data may be affected by social desirability. Social desirability effects have been reported to be limited in traffic-related studies, and the anonymous completion of the online survey may have helped to reduce such effects [60]. Moreover, previous studies testing the links between driver attributes and behavior toward cyclists using self-reporting [22] or simulation-based data [61] show similar results. The levels of self-reported aggressive behavior toward cyclists were overall low in the present study. Importantly, while the variance may have been limited in the outcome variable, the data show effects that explain variation in the outcome. Future research can improve on the self-reported data used here by considering new technologies to collect information on car driver and cyclist interactions (e.g., BikeMaps.org [62] as well as bicycle use more generally [63]).

## 5. Conclusions

This research provides insights into the ways in which perceived norms regarding driver behavior and sustainability are linked to aggressive driver behavior toward cyclists. Norms observed in the road context, and which are directly related to the targeted behavior, are well-placed to inform aggressive behavior toward cyclists. Norm interventions can effectively address driver aggressive behavior toward cyclists by targeting norms regarding this specific behavior. Our findings suggest that such interventions can be complemented with interventions targeting norms perceived in workplaces (or other road adjacent contexts) and related to sustainability (green psychological workplace climate), in road contexts where drivers perceive aggression toward cyclists to be common. Such interventions are especially relevant in relation to the type of aggressive warnings targeted in this study as laws are ill-suited to regulate them.

## Figures and Tables

**Table 1:** Correlations and descriptive statistics.

	Mean	*SD*	1	2	3	4	5	6	7	8
1. Gender	0.49	0.50								
2. Cycle frequency (past 12 months)	4.23	1.61	−0.28 **							
3. Time driver’s license held	26.59	13.59	0.17 **	−0.24 **						
4. Job tenure	9.99	9.10	0.19 **	−0.11 *	0.51 **					
5. Full-time employment	0.68	0.47	0.34 **	−0.13 **	0.01	0.10 *				
6. Negative attitudes toward cyclists	41.91	12.30	−0.08	0.58 **	−0.18 **	−0.01	−0.01			
7. Perceived norms regarding aggressive driver behavior toward cyclists	2.60	0.76	−0.02	−0.07	−0.12 *	−0.12 *	0.02	0.00		
8. Perceived green psychological workplace climate	3.75	0.91	0.02	−0.11 *	−0.01	−0.02	0.07	−0.06	−0.05	
9. Aggressive driver behavior toward cyclists	1.20	0.40	0.09	0.16 **	−0.18 **	−0.12 *	0.05	0.36 **	0.38 **	−0.07

Note: ** *p* < 0.01; * *p* < 0.05 (2-tailed); participant gender coded so that 1 = male and 0 = female; cycling frequency coded so that higher values represent lower cycling frequency; full-time employment coded so that 0 = part-time work; 1 = full-time work.

**Table 2:** Regression analysis of aggressive driver behavior toward cyclists on norm perceptions.

Variable		
	*ß*	*SE*
Step 1		
Gender	0.13 *	0.04
Cycle frequency (12 months)	−0.06	0.02
FTE	0.00	0.04
Job tenure	−0.10	0.00
Length of license	−0.10	0.00
Negative attitudes toward cyclists	0.38 **	0.00
* R* ^2^	0.165	
*F* (6, 404) = 13.34; *p <* 0.000		
Step 2		
Gender	0.13	0.04
Cycle frequency (12 months)	−0.01	0.01
FTE	0.00	0.04
Job tenure	−0.06	0.00
Length of license	−0.07	0.00
Negative attitudes toward cyclists	0.36 **	0.00
Perceived norms of aggressive driver behavior toward cyclists	0.37 **	0.02
Perceived green psychological workplace climate	−0.03	0.02
* R* ^2^	0.30	
Δ*R*^2^	0.14	
*F* (8, 402) = 21.98; *p* < 0.001		

Note: ** *p* < 0.000; * *p* < 0.005; participant gender coded so that 1 = male and 0 = female; cycling frequency coded so that higher values represent lower cycling frequency; full-time employment coded so that 0 = part-time work; 1 = full-time work.

**Table 3:** Interaction of perceived norms of aggressive driver behavior toward cyclists and perceived green psychological workplace climate.

	*Effect*	*SE*	CI 95%
Gender	0.11	0.04	0.03/0.18
Cycle frequency (12 months)	0.00	0.01	−0.03/0.02
FTE	0.01	0.04	−0.07/0.08
Job tenure	0.00	0.00	−0.01/0.00
Length of license	0.00	0.00	−0.01/0.00
Negative attitudes toward cyclists	0.01	0.00	0.01/0.01
Perceived norms of aggressive driver behavior toward cyclists	0.45	0.09	0.29/0.62
Perceived green psychological workplace climate	0.17	0.06	0.05/0.30
Interaction term	−0.07	0.02	−0.11/−0.03
*R* ^2^	0.32		
*F* (9, 401) = 21.06; *p* = 0.000			

Note: participant gender coded so that 1 = male and 0 = female; cycling frequency coded so that higher values represent lower cycling frequency; full-time employment coded so that 0 = part-time work; 1 = full-time work.

## Data Availability

The participants of this study did not provide written consent for their data to be shared publicly, so due to the sensitive nature of the research supporting data are not available.

## Supplementary Materials

The following supporting information can be downloaded at: https://www.mdpi.com/article/10.3390/ijerph20065163/s1, Table S1: Detailed overview of sample characteristics.
