# Supplementary material for: Why Not Pedal for the Planet? The Role of Perceived Norms for Driver Aggression as a Deterrent to Cycling
Source: Int J Environ Res Public Health. 2023 Mar 15;20(6):5163. doi: 10.3390/ijerph20065163 (PMC10049320; doi:10.3390/ijerph20065163)
Supplement: Supplementary file 1 [file ijerph-20-05163-s001.zip › ijerph-2252669-supplementary.pdf]

Table S1
Detailed breakdown of sample characteristics

Sample characteristics (N= 426)	Frequencies and descriptives
**Demographics**	
Age (years)	M = 43.59<br>SD= 13.59<br>min = 18<br>max = 82
Gender	
Male	206 (49.3%)
Female	212 (50.7%)
Education	
Some High School	14 (3.3%)
Completed High School	41 (9.6%)
Vocational Education/Diploma	90 (21.1%)
Currently enrolled in a Bachelor degree	22 (5.2%)
Completed Bachelor degree	135 (31.7%)
Currently enrolled in postgraduate studies	29 (6.8%)
Completed Masters degree	69 (16.2%)
Completed Doctorate	26 (6.1%)
**Work related characteristics**	
Full time vs part time employment	
Full time	285 (67.7%)
Part time	136 (32.3%)
Job tenure	M = 9.99<br>SD = 9.06<br>min = 1<br>max = 49
Industry	
Education and training	77 (18.2%)
Professional, scientific and technical services	56 (13.2%)
Other	52 (12.3%)
Health care and social assistance	47 (11.1%)
Mining	30 (7.1%)
Administrative and support services	26 (6.1%)
Transport, postal and warehousing	24 (5.7%)
Retail trade	20 (4.7%)
Construction	16 (3.8%)
Public administration and safety	15 (3.5%)
Agriculture	14 (3.3%)
Accommodation and food services	13 (3.1%)
Information media and telecommunications	8 (1.9%)

Arts and recreation services	8 (1.9%)
Rental, hiring and real estate services	7 (1.7%)
Manufacturing	5 (1.2%)
Electricity, gas, water and waste services	4 (0.9%)
Wholesale trade	2 (0.5%)
**Transport related characteristics**	
Time driver's license held (years)	M = 25.59
	SD = 13.59
	min = 1
	max = 65
Cycling frequency (in the past 12 months)	
Every day	17 (4.0%)
Most days	71 (16.7%)
Once or twice a week	59 (13.8%)
Once or twice a month	71 (16.7%)
Once or twice in the year	68 (16.0%)
Never	140 (32%)
